# Urethral Complications Due to Erythema Multiforme Major: A Case Report

**DOI:** 10.7759/cureus.76268

**Published:** 2024-12-23

**Authors:** Zachary A Dwyer, Tanner Shields, Jackson Parks, Benjamin Byler, Baydon E Hilton, Jeremy J Houser

**Affiliations:** 1 Department of Anatomy, A.T. Still University of Health Sciences - Kirksville College of Osteopathic Medicine, Kirksville, USA; 2 Department of Dermatology, Northeast Regional Medical Center, Kirksville, USA

**Keywords:** emm, erythema multiforme major, male urethra, wound care modalities, wound care techniques

## Abstract

Erythema multiforme major (EMM) is an acute, immune-mediated mucocutaneous disease that rarely affects the genital mucosal surfaces. This study describes a 39-year-old male with this rare disease and unusual presentation. The patient presented to an emergency department with oral lesions, drainage from both eyes, injected sclera, and characteristic targetoid lesions on the face, upper extremities, torso, and plantar surfaces of the feet. Laboratory results indicated a latent herpes simplex virus type 1 (HSV-1) infection and an active *Mycoplasma pneumonia* infection. The patient later developed dysuria and sloughing of the urethral mucosa, which was managed conservatively with topical lidocaine and petroleum-impregnated gauze. This treatment resulted in full healing and return of function. Currently, there is limited guidance on treating genital manifestations of EMM. Although this disease is rare, protocols should be developed for the treatment and care of future patients.

## Introduction

Erythema multiforme (EM) is an acute, immune-mediated mucocutaneous disease characterized by targetoid erythematous lesions that often have an acral localization [1]. The incidence of this rare disease is estimated to be one to two cases per million person-years, and it most commonly occurs in adults under 40 years of age [2,3]. Although its exact mechanism is unclear, EM is considered a type IV hypersensitivity reaction mediated by T-cell response to viral, bacterial, or chemical antigen stimulation that causes epidermal damage [4,5]. The most common identifiable cause of EM is herpes simplex virus type 1 (HSV-1); however, numerous other etiologies have been reported, including *Mycoplasma pneumoniae*, Epstein-Barr virus, hepatitis C virus, coxsackievirus, and pharmacologic agents, such as sulfonamides, penicillins, statins, or nonsteroidal anti-inflammatory drugs (NSAIDs) [2,4,6-8]. A subclassification of EM, erythema multiforme major (EMM), is characterized by mucous membrane and systemic involvement that commonly occurs in the oral mucosa [8]. In rare cases, ocular and genital mucosal lesions can also occur [6,7]. This study describes a 39-year-old male diagnosed with EMM who subsequently developed genital mucosal lesions.

## Case presentation

**Figure 1:**
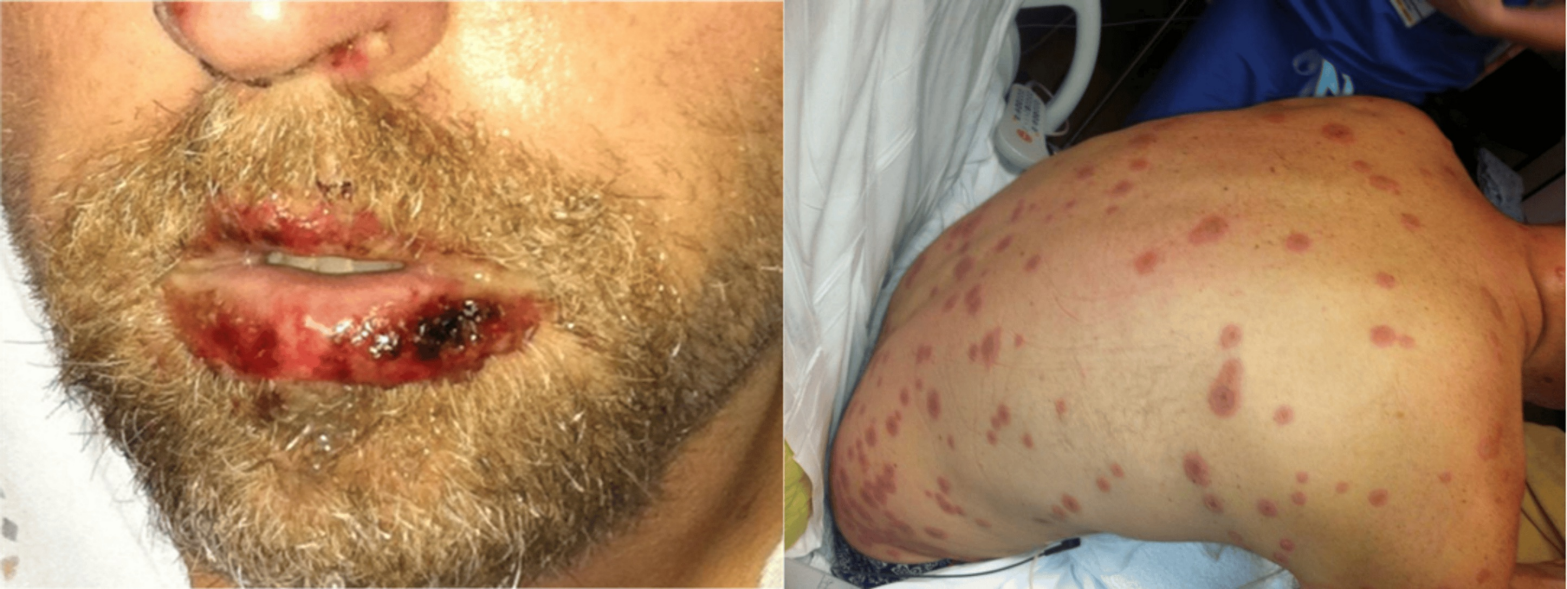
EMM lesions in the patient. The images show EMM with mucosal involvement. On the left, labial mucosal lesions and sloughing secondary to EMM are seen. On the right, targetoid lesions, commonly observed in EMM, are seen. EMM: erythema multiforme major

A 39-year-old male presented to the emergency department (ED) with generalized fatigue, an erythematous rash, oral lesions, and drainage from both eyes (Figure 1). He reported general symptoms of not feeling well including sore throat, chest congestion, night sweats, and a productive cough since the beginning of the previous week and had been taking ibuprofen daily. The patient noted exposure to a sick contact with a similar febrile illness a week before the onset of symptoms. Prior to presenting to the ED, he developed a fever, painful oral lesions, conjunctivitis, and 5 mm targetoid lesions on his face, upper extremities, chest, and plantar surfaces of both feet. On initial examination, the patient was alert with mild tachycardia and mild tachypnea, but his vitals were not overtly concerning. Conjunctivitis with injected sclera and clear drainage was observed bilaterally; there were also vesicular perioral lesions with sloughing of both oral labial and the oropharyngeal mucosa. The patient was admitted to the hospital, and ophthalmology and dermatology were consulted. The dermatologic and ophthalmologic treatment of this patient's case has been previously described [9].

The initial laboratory results are shown below but serology results indicated an active infection with *M. pneumoniae* and an underlying infection with HSV-1 (Table 1). Based on the clinical presentation, inflammatory markers, and positive serologies, the patient was diagnosed with EMM. Initial management included intravenous fluid supplementation. The patient was then started on a five-day course of azithromycin (500 mg), a seven-day course of acyclovir (700 mg), and daily intravenous methylprednisolone (40 mg). The pain was well controlled with patient-controlled fentanyl analgesia. Oral lesions were managed with the application of 10 mL of viscous lidocaine gel and 20% benzocaine spray as needed. Ocular mucosal lesions were treated with artificial tear ophthalmic ointment and Lacri-Lube eye ointment application every 4 hours. Skin lesions were covered with petroleum jelly and monitored daily.

**Table 1:** Initial laboratory results. HSV: herpes simplex virus

Lab test	Test value	Normal range
Erythrocyte sedimentation rate	60 mm/h	<15 mm/h
C-reactive protein	18.6 mg/dL	<0.3 mg/dL
*M. pneumoniae* IgM	1.94 U/L	<0.76 U/L
*M. pneumoniae* IgG	2.24 U/L	<0.09 U/L
HSV-1 IgM	0.82 index	<0.9 index
HSV-1 IgG	5.27 index	<0.89 index

**Figure 2:**
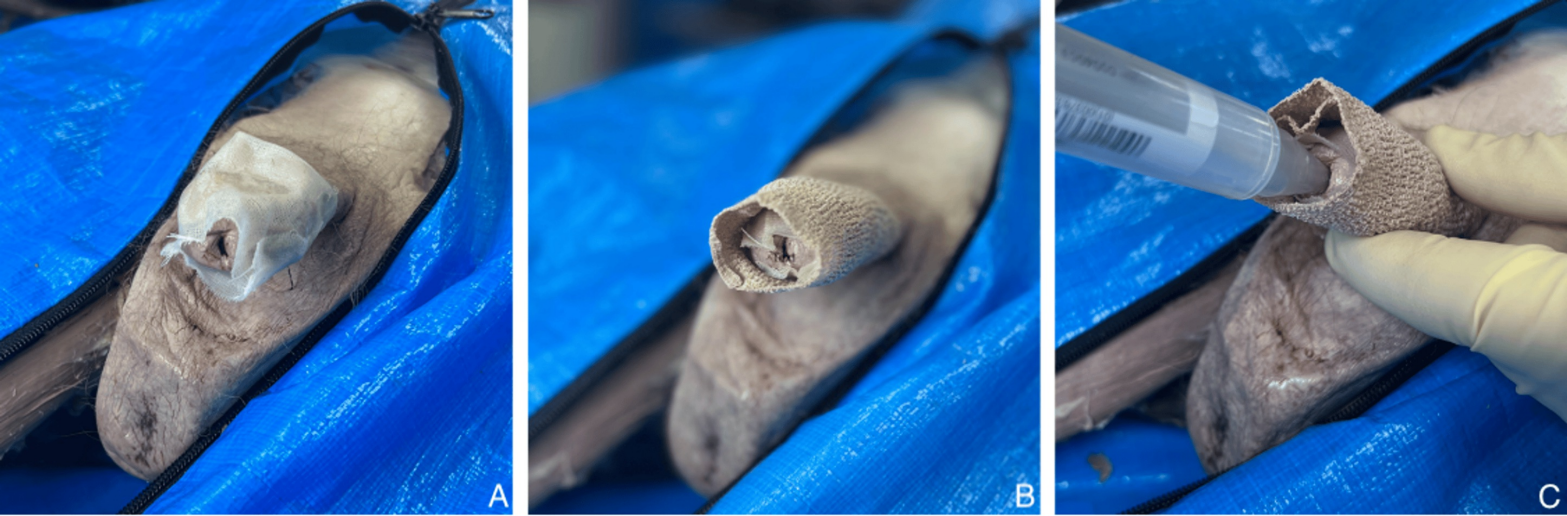
Treatment of genital mucosal lesions. Treatment of genital mucosal surfaces in the present case, as demonstrated on a cadaver penis. (A) Application of the petroleum-impregnated gauze wrap. (B) Application of the self-adhesive elastic bandage to cover the gauze. (C) Administration of the topical lidocaine injector after the dressings were applied.

Within 36 hours of hospitalization, the patient developed dysuria. Examination indicated erosions and sloughing of urethral mucosa involving the urethral meatus and blistering of the glans penis, which is an unusual complication of EMM. Dysuria and slow wound healing were problematic throughout hospitalization because the denuded mucosa adhered to the hospital gowns and linens. Initially, wounds were dressed with dry gauze and tape; however, after discharge from the hospital, they were switched to petroleum-impregnated gauze and self-adhesive bandages (Figures 2A-2C). This petroleum-impregnated gauze was wrapped loosely around the distal half of the penile shaft with the open end of the gauze extending past the glans by a few centimeters (Figure 2A). The self-adherent elastic wrap was then wrapped around the petroleum-impregnated gauze and the proximal half of the penile shaft to maintain the gauze position (Figure 2B). Urethral pain was initially managed with a 5-mL lidocaine URO-Jet (South El Monte, CA: International Medication Systems, Ltd.) 2% intraurethral applicator (Figure 2C). This gel was directly applied to the surface of the external urethral meatus and glans penis. After a 10-second pause to allow for anesthesia of the meatus, the applicator was pushed through the adhesion at the orifice and a bolus of the lidocaine gel was injected into the distal urethra, allowing for frequent urination with minimal pain. At the time of hospital discharge, the patient was still experiencing dysuria, urethral mucosal sloughing, and lesions on the glans penis. However, urethral injury resolved over several weeks following treatment with a prednisone taper, oxycodone, and conservative wound management.

## Discussion

The present study described the diagnosis and treatment of a 39-year-old male diagnosed with EMM who developed a rare complication that affected genital mucosal surfaces. Historically, a diagnosis of EM or Stevens-Johnson syndrome has been combined into a single diagnosis; however, Bastuji-Garin et al. proposed that EM, Stevens-Johnson syndrome, and other bullous skin diseases are separate entities that can be differentiated based on clinical presentation [10]. For example, EM is characterized by the detachment of skin that affects less than 10% of the body surface area and by the presence of typical or atypical erythematous targetoid lesions that may have a central bullae formation [2]. Stevens-Johnson syndrome also affects less than 10% of body surface area, but it forms erythematous macules or atypical target lesions that progress to areas of erythema with dusky centers, flaccid blisters with a positive Nikolsky sign, and sheets of denuded epidermis [11]. This syndrome is associated with penicillin and sulfonamide use, while EM is more commonly associated with viral and bacterial infections [1].

Clinically, EM can be subclassified as erythema multiforme minor, which has no mucosal involvement, or erythema multiforme major, which has mucosal membrane involvement and systemic symptoms. Oral mucosal involvement is by far the most common manifestation of EMM, but lesions can also be present in ocular and genital mucosa in rare cases [8]. Lesions that involve genital mucosa occur in about 15-25% of EMM cases [6,7]. In the present case, the patient first developed a febrile illness followed by epidermal lesions characteristic of EM. However, those symptoms rapidly progressed to involve the oral labial mucosa, buccal mucosa, oropharyngeal mucosa, ocular conjunctiva, glans penis, and urethral meatus, culminating in this rare presentation of EMM.

Research suggests that the pathogenesis of EM is driven by an inflammatory cascade that results in the recruitment of autoreactive cytotoxic T-cells, which cause keratinocyte apoptosis and growth arrest [12]. Histopathologic features of this disease include lymphocytic infiltrate at the epidermis-dermis interface, lymphocytic attachment to necrotic keratinocytes, and papillary dermis edema [1]. Although EM can be precipitated by various antigens, HSV-1 is the most common cause of EMM and the most common cause of recurrent EMM [2,7,13]. Other infectious agents, such as *M. pneumoniae* and hepatitis C, or pharmacologic agents, such as NSAIDs or sulfonamides, can also trigger EMM [2]. Based on the bacterial and viral serology results, the patient was found to have an active *M. pneumoniae* infection and a latent HSV-1 infection. He also reported recent use of NSAIDs for antipyresis during the days before he presented to the emergency department. Based on the patient’s recent contact with a sick person and his general malaise, pulmonary symptoms, and bacterial serology, it would be reasonable to attribute his fever to *M. pneumoniae*. However, the exact etiology of his EMM diagnosis is unknown because there were multiple contributing variables.

The treatment of EM varies depending on the extent of the disease, severity of the lesions, and underlying etiological agent. Currently, there is no widely accepted standard of care for the treatment of EM, and recommendations are largely based on expert opinion and small retrospective case series [8]. Because genital mucosal involvement is a rare presentation of EMM, there is even less data to support an optimal treatment. Current recommendations suggest removal of the offending agent as the initial step for the successful management of EM [14]. In cases of drug-induced EM, administration of the pharmacological agent should be stopped immediately. Because most cases of EM are preceded by or coincide with an infection, identification of the etiological organism and administration of the appropriate antimicrobial pathogen should be the first-line treatment, especially if the patient has a symptomatic infection [8]. However, there is evidence indicating that the use of anti-HSV medications does not alter the course of acute EM eruptions [14].

Another goal for the management of EM is to decrease the severity and duration of the acute inflammatory response. Minimal mucosal involvement is often treated with topical corticosteroids and topical anesthetics [1]. In cases of severe mucosal involvement, systemic glucocorticoids are often used. Although monthlong prednisone tapers (20-60 mg/day tapered over four weeks) have been suggested, there are no controlled studies to support this recommendation [14]. To decrease the pain and inflammation of mucosal lesions, topical glucocorticoids and anesthetic solutions are recommended [8]. For the patient in the present case, all home medications were withheld, including the ibuprofen he had previously taken for fever and 40 mg of methylprednisolone he was administered intravenously. After confirmation of current *M. pneumoniae* infection, he was given azithromycin and HSV-1 was also empirically covered with acyclovir due to latent infection.

The unique aspect of this case was the extent and severity of mucosal involvement of oral, ocular, and genital mucosal surfaces during the patient’s acute EMM eruption. The specific management of the ocular mucosal lesions was reported by Senger et al. [9]. For the current report, we focused on uncommon genital manifestations, which can cause marked pain and discomfort. Because genital involvement is rare, long-term complications of EMM lesions on genital mucosal surfaces are generally unknown. A study from 1981 reported vaginal stenosis as a complication of bullous EM, but no studies were found reporting similar complications in males [15]. In the present case, the patient reported symptoms of dysuria several days after being admitted to the hospital. He had developed painful bullae of the glans penis and mucosal sloughing with erosions of the distal urethral epithelium. Wound healing of the external glans lesions was slow because of the weeping nature of the bullae, which tended to stick to the hospital gown or dry gauze and caused additional discomfort and tissue damage when removed. For example, when the dressing was changed to allow for urination, the bullae would often tear open. After discharge from the hospital, the dry gauze and medical tape were modified to a petroleum-impregnated gauze with a self-adherent elastic bandage wrap (Figures 2A-2C). This dressing arrangement had numerous benefits. The petroleum-impregnated gauze minimized the adhesion of the gauze to the bullae and allowed for urination without a dressing change because of its hydrophobic properties. The open end of this dressing also allowed the application of the topical lidocaine gel for pain management (Figure 2C).

Another complication of the present case was also related to the slowed wound healing of the mucosa of the distal urethral epithelium. The repeated adhesion and separation cycle that occurred because of urination delayed the healing process. When empty, the urethra is naturally a flattened tube, and the adjacent walls of the denuded urethra tend to adhere. During micturition, the pressurized urine tears the adhesions to achieve flow through the urethra. This caused the patient intense pain with urination, and the administration of intravenous fluids for hydration during his hospitalization increased urinary output. Acutely, this symptom was managed with topical lidocaine gel that was directly applied to the surface of the external urethral meatus and glans penis. This treatment allowed frequent urination with minimal pain. After hospital discharge, these genital lesions fully healed by secondary intention over the next several weeks with no urethral strictures or lasting complications.

## Conclusions

Erythema multiforme major is a rare, self-limiting disorder involving mucosal surfaces. Healthcare providers need to be aware that genital involvement is an uncommon complication of EMM that requires careful treatment of lesions of the glans and the urethra. Currently, there is limited guidance on the treatment of genital manifestations of EMM. However, the treatment methods used in the present case of genital mucosal lesions - petroleum-impregnated gauze, self-adhesive elastic wrap, and a topical lidocaine gel applicator - had positive outcomes and should be considered for future cases. Even though these complications are relatively rare in patients diagnosed with EMM, a set of guidelines should be developed to facilitate the treatment and care of future patients.

## References

[1] Roujeau J (2012). Erythema multiforme. Fitzpatrick's Dermatology in General Medicine. Eighth Edition.

[2] Lerch M, Mainetti C, Beretta-Piccoli BT, Harr T (2018). Current perspectives on erythema multiforme. Clin Rev Allergy Immunol.

[3] Huff JC, Weston WL, Tonnesen MG (1983). Erythema multiforme: a critical review of characteristics, diagnostic criteria, and causes. J Am Acad Dermatol.

[4] Sanchis JM, Bagán JV, Gavaldá C, Murillo J, Diaz JM (2010). Erythema multiforme: diagnosis, clinical manifestations and treatment in a retrospective study of 22 patients. J Oral Pathol Med.

[5] Chrysomali E, Lozada-Nur F, Dekker NP, Papanicolaou SI, Regezi JA (1997). Apoptosis in oral erythema multiforme. Oral Surg Oral Med Oral Pathol Oral Radiol Endod.

[6] Schofield JK, Tatnall FM, Leigh IM (1993). Recurrent erythema multiforme: clinical features and treatment in a large series of patients. Br J Dermatol.

[7] Wetter DA, Davis MD (2010). Recurrent erythema multiforme: clinical characteristics, etiologic associations, and treatment in a series of 48 patients at Mayo Clinic, 2000 to 2007. J Am Acad Dermatol.

[8] Trayes KP, Love G, Studdiford JS (2019). Erythema multiforme: recognition and management. Am Fam Physician.

[9] Senger B, Memar SA, Ahmann A, Houser JJ, Doughty-McDonald L (2021). Dermatologic and ophthalmologic treatment of erythema multiforme major: a case report. Cureus.

[10] Bastuji-Garin S, Rzany B, Stern RS, Shear NH, Naldi L, Roujeau JC (1993). Clinical classification of cases of toxic epidermal necrolysis, Stevens-Johnson syndrome, and erythema multiforme. Arch Dermatol.

[11] Frantz R, Huang S, Are A, Motaparthi K (2021). Stevens-Johnson syndrome and toxic epidermal necrolysis: a review of diagnosis and management. Medicina (Kaunas).

[12] Aurelian L, Ono F, Burnett J (2003). Herpes simplex virus (HSV)-associated erythema multiforme (HAEM): a viral disease with an autoimmune component. Dermatol Online J.

[13] Schalock PC, Dinulos JG, Pace N, Schwarzenberger K, Wenger JK (2006). Erythema multiforme due to mycoplasma pneumoniae infection in two children. Pediatr Dermatol.

[14] Sokumbi O, Wetter DA (2012). Clinical features, diagnosis, and treatment of erythema multiforme: a review for the practicing dermatologist. Int J Dermatol.

[15] Graham-Brown RA, Cochrane GW, Swinhoe JR, Sarkany I, Epsztejn LJ (1981). Vaginal stenosis due to bullous erythema multiforme (Stevens-Johnson syndrome). Br J Obstet Gynaecol.

